# Effectiveness of Workplace Yoga Interventions to Reduce Perceived Stress in Employees: A Systematic Review and Meta-Analysis

**DOI:** 10.3390/jfmk5020033

**Published:** 2020-05-26

**Authors:** Elisabetta Della Valle, Stefano Palermi, Irene Aloe, Roberto Marcantonio, Rocco Spera, Stefania Montagnani, Felice Sirico

**Affiliations:** Department of Public Health, University of Naples “Federico II”, 80131 Naples, Italy; elisabetta.dellavalle@unina.it (E.D.V.); stefano.palermi@unina.it (S.P.); aloeirene@gmail.com (I.A.); roberto.marcantonio@unina.it (R.M.); rocco.spera@unina.it (R.S.); montagna@unina.it (S.M.)

**Keywords:** worksite, corporate wellness programs, workers, complementary medicine, relaxation

## Abstract

Work-related stress represents a relevant public health issue and solution strategies are mandatory. Yoga is a common approach to manage stress and its effectiveness has been extensively confirmed. Therefore, this study aims systematically to review the effectiveness of Yoga interventions carried out at workplace on work-related stress among employees and to assess their impact quantitatively. Springerlink, MEDLINE, PubMed, CINAHL, Web of Science, Scopus, Cochrane CENTRAL and PEDro databases were searched. Clinical trials comparing workplace Yoga interventions to control groups, and evaluating perceived stress as outcome measure, were assessed for eligibility. All forms and styles of Yoga were considered for the analysis. Out of 3392 initially identified, 6 studies were included in the meta-analysis; 266 participants practicing Yoga interventions at worksite were compared to 221 subjects in control group. Included studies showed “some concerns” about different domains of source of bias. Quantitative analysis showed an overall effect size of −0.67 [95% confidence interval (CI): −0.86, −0.49] in favor of Yoga intervention in reducing stress outcome measures. Hence, workplace Yoga interventions were more effective when compared to no treatment in work-related stress management. Further high-quality studies are needed to improve the validity of these results and to specify more characteristics of the Yoga intervention, such as style, volume, and frequency.

## 1. Introduction

Work-related stress can be defined as an emotional consequence occurring when the workplace demand exceeds the worker’s ability to cope with it, causing potential detrimental physical effects [1]. It has a relevant role in the onset of cardiovascular [2], musculoskeletal [3] and mental health disorders [4], as previously demonstrated in literature.

A recent statement of the European Agency for Safety and Health at Work [5] highlights a worrying scenario about this topic. Indeed, work-related stress is identified as a widespread pathology with a huge economic burden, mainly explained by loss of productivity and direct and indirect related sanitary assistance. Its incidence is even higher than the incidence of musculoskeletal problems among workers: 600,000 vs. 500,000 new or longstanding cases, with a prevalence rate of 1800 per 100,000 workers [1,5]. A total of 12.8 million working days are lost due to work-related stress, depression, or anxiety, with a mean value of 21.2 days lost per case [5]. Obviously, these data highlight a huge economic impact associated to this condition, beyond health aspects.

Many strategies have been developed to prevent and to treat this disease [6]. As an example, changes in lifestyles represent a cornerstone in the prevention of some non-communicable diseases, including stress. Promoting healthy nutritional approaches [7,8] and fighting physical inactivity [9] have also shown effective results in stress prevention and management, even in different work environments [10,11].

In order to promote the participation of employees to several health programs carried out directly at workplace, several corporate wellness programs have been implemented over the years [12]: among these, some are extended to the employees’ families too, including partners and sons [12]. Part of corporate wellness programs include medical activities exclusively, such as cardiovascular check-up, dermatologic check-up or some other specialist medical consultations carried out all over the year [13]. Some others include lifestyle interventions such as tobacco cessation strategies, dietary improvement through food selection in restaurants, reduction of screen time and sitting time, and physical activity programs [14,15].

In most advanced realities [12], lifestyle and medical programs are organized together: some specific spaces within the company are dedicated to employees (like gym or open spaces to practice physical activity); an on-site clinic is available for medical consultations and diagnostic activities and others structured interventions are proposed to employees. Several activities to reduce the work-related stress are often included in these programs, confirming the relevance of this topic among employees [13]. Meditation strategies are gaining growing scientific interest [16,17] as an effective stress-management intervention., Among these, a simple and effective approach is represented by Yoga interventions.

Yoga is a form of mind-body fitness that involves a combination of muscular activity and an internally mindful training [18]; it is classified by the National Institutes of Health as a form of Complementary and Alternative Medicine [19]. The relationship between physical and mental health is well described in literature [20], and this is a topic investigated among workers [21]. It has been previously demonstrated [22] the effectiveness of Yoga for stress management, with an overall improvement of quality of life (QoL). Previous studies [23,24] have proved the effectiveness of Yoga in changing some physiological parameters associated to stress, like hematic level of cortisol, reduction of systolic blood pressure and heart rate. Probably these results are due to some psycho-neuro-endocrine-immune mechanisms, determining an improving in parasympathetic activity, optimizing secretion of sympathetic hormones and reducing metabolic rate [25] 

Although these quantitative data showed positive effects of Yoga on some health parameters in general population, few evidences analyzed the effect of Yoga interventions at workplace on perceived stress directly. While a previous review [26] reported the general effectiveness of Yoga for healthy adults, to the authors’ best knowledge no systematic review has investigated the effectiveness of Yoga interventions conducted at workplace, as part of corporate wellness program, on work-related stress reduction.

Therefore, the purpose of this systematic review and meta-analysis is to assess the effectiveness of Yoga interventions conducted at workplace in reducing perceived stress among employees.

## 2. Materials and Methods

### 2.1. Data Sources and Searches

**Figure 1:**
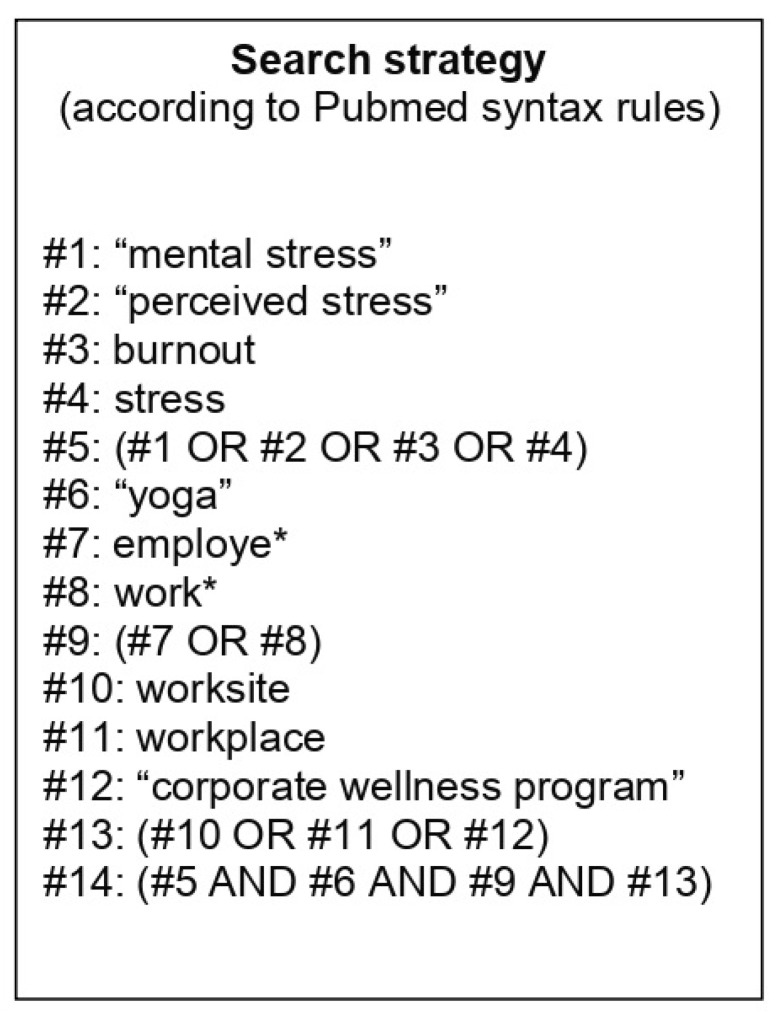
Detailed search strategy for Pubmed.

A systematic review of the available literature was performed and reported according to PRISMA (Preferred Reporting Items for Systematic Reviews and Meta-Analyses) guidelines [27]. For the study, the authors used the following databases, from each database’s inception until May 2020: Springerlink, MEDLINE, PubMed, CINAHL, Web of Science, Scopus, Cochrane CENTRAL and PEDro. Some keywords were used to build a research key for the main topic of the study like “stress”, “Yoga”, “workplace” and others. The complete research strategy for one of the databases used (Pubmed) is reported in Figure 1. Each database was searched according to its specific syntax rules. Moreover, manual search of published and unpublished studies (conference abstracts, textbooks, “grey” literature) was also conducted and reference lists of retrieved articles were screened.

### 2.2. Study Selection

To try to obtain the highest level of evidence, the analysis has been limited to randomized and non-randomized controlled trials. English language restriction was applied. Observational studies, case reports and other study designs of less methodological rigor and without a control group have been excluded.

The main inclusion criteria were: (i) Yoga interventions carried out at workplace, (ii) randomized and non-randomized study design with at least two arms of intervention (Yoga vs. control), (iii) at least one measure of perceived stress as a dependent variable of the study. Although several forms of Yoga exist, the main characteristics of flexibility, coordination, breathing, and gentle movements are similar. No reliable data are available to define a higher effectiveness of a specific form of Yoga compared to the others about stress reduction and other outcomes. Therefore, any form of Yoga intervention has been considered eligible for the purpose of the present study. If a study reported more than one outcome measures for perceived stress, through different methods or evaluation procedures, all these data have been included in the quantitative analysis.

After the removal of duplicates, titles and abstracts of retrieved articles were screened by two independent researchers (S.P. and I.A.) to assess for eligibility. Papers selected by the two researchers were compared and eventual disagreements were solved by discussion with a third author (E.D.V.). Subsequently, full texts of selected articles were retrieved.

### 2.3. Data Extraction and Quality Assessment

A specific extraction data form was designed for this study. The following data were extracted from each full text: authors’ name, year and country of publication, total sample size, number of subjects in each treatment arm, characteristics of the Yoga intervention carried out, length of the intervention, main outcome measures of perceived stress, relevant clinical findings.

The studies included in the analysis were assessed for risk of bias according to their study design. Randomized clinical trials have been assessed by the revised Cochrane risk-of-bias tool (RoB 2.0) [28]. The following domains of bias were considered: randomization process, deviations from the intended interventions, missing outcome data, measurement of the outcome and selection of the reported result. The overall risk of bias of each study was considered “low”, “high” or affected by “some concerns”. Non-randomized studies of intervention have been analyzed by the risk of bias in non-randomized studies of interventions assessment tool (ROBINS-I) [29], assessing for confounding, selection of participants, classification of interventions, deviations from intended interventions, missing data, selection of the reported result and giving an overall judgment about risk of bias (low, moderate, serious, critical). Data extraction procedures and risk of bias assessment of included studies were conducted by two authors (R.M. and E.D.V.) separately. Results were paired, and disagreements were solved by discussion with a third author (F.S.).

### 2.4. Statistical Analysis

The extracted data were analyzed using STATA software (StataCorp. v.12, College Station, TX, USA), through the metan routine. Effect sizes and 95% confidence intervals were calculated for each study according to data types. Included studies used different outcome measures for perceived stress and the summary effect was assessed by standardized mean difference (SMD) according to Borestein et al. [30]. Based on the characteristics of included studies, summary estimates of effect were calculated with a random-effects model. Included studies, according to the previous inclusion criteria, had to report at least one outcome measure of stress. The stress assessment was conducted pre- and post-intervention in both groups (Yoga and Control), namely at baseline and at the end of the experimental phase. The mean difference and standard deviation of parameters from pre-intervention and post-intervention were calculated according to previous described methods [30].

In studies reporting data as mean and standard error, the standard deviations were calculated multiplying standard error for square root of numbers of subjects included in the correspondent arm of treatment, to include these data in the quantitative analysis.

Some outcome scales adopted in included studies had different directions in their interpretation. For example, lower scores in the Perceived Stress Scale (PSS) indicate a low stress degree. On the other hand, higher scores in the Inventory of Positive Psychological Attitudes Scale (IPPA) and in the Stress Adaptation Scale (SAS) indicate a better adaptation to stress. Therefore, data assessed through these scales were arranged coherently to the direction of the effect to permit quantitative analysis.

*I^2^* was used to express heterogeneity as percentage. Significant heterogeneity was considered if an *I^2^* > 60% was recorded.

## 3. Results

### 3.1. Search Results

**Figure 2:**
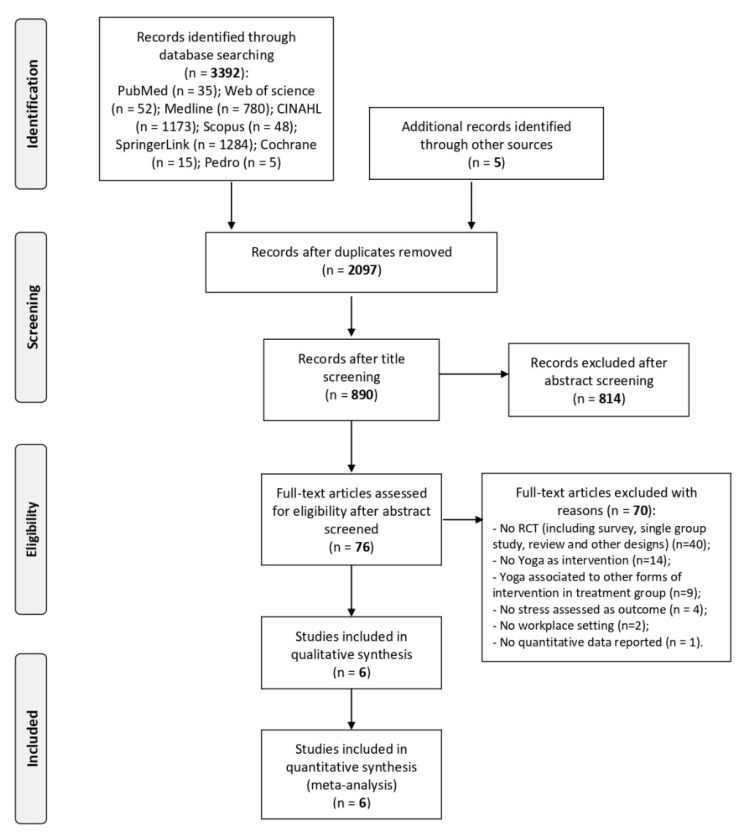
Flow chart of studies search and selection, according to Preferred Reporting Items for Systematic Reviews and Meta-Analyses (PRISMA).

Out of 3392 articles initially identified, titles and abstracts of 890 studies were screened after removal of duplicates. Based on inclusion and exclusion criteria, 76 studies exploring the effect of Yoga interventions carried out at workplaces on the reduction of stress were considered for a detailed evaluation, and their full texts were retrieved. Among them, 70 were excluded for the following reasons: 40 were not randomized or non-randomized controlled trials (including survey, single-arm intervention study without control group, narrative review about the topic and other designs); 14 did not conduct Yoga as an intervention but other programs that were not completely specified; 9 studies used Yoga in addition to other interventions in the treatment group; 4 studies did not assess stress as an outcome directly; 2 studies were conducted in settings different from workplaces; and 1 study did not report sufficient quantitative data to be included in qualitative synthesis and quantitative analysis. Figure 2 shows the flow chart of the study search and selection 2.

### 3.2. Study Characteristics

Trials included in the analysis enrolled a total of 487 patients: 266 in the treatment group and 221 in the control group. Specific data about gender of patients was not reported in Axén et al. [31], and Wolever et al. reported this data as a percentage [32]; nevertheless, a higher percentage of females were included in the sample population of almost all included studies. The mean age of participants ranged from 32.07 ± 7.54 years in Lin et al. [33] to 54 ± 8.1 in Axén et al. [31].

All included studies had a two-arm design, comparing a Yoga program to a control group (no activity). Only Wolever et al. [32] had a more complex design. Indeed, in this multicentric study a Yoga intervention was compared to a mindfulness program (carried out at workplaces and through an online platform) and to a control group. Both interventions (Yoga and mindfulness programs) were effective in reducing perceived stress compared to control group. Only data comparing Yoga to a control group were used to the purpose of the present study, according to inclusion criteria.

Axén et al. [31] and Hartfiel et al. [34] added extra-sessions of Yoga intervention performed at home, beyond interventions carried out at workplaces.

Included studies reported data from Yoga interventions carried out at workplaces, among different kind of employees. Axén et al. [31] and Lin et al. [33] included health personnel as their study population. Other studies included employees of a telephone industry [35], university employees [34], local government authority employees [36], and employees of a national insurance carrier [32].

The PSS was adopted as the outcome scale by three out of six included studies [31,32,36]; Bhandari et al. [35] adopted the Cornel Medical Index Health Questionnaire (CMIHQ), Hartfiel et al. [34] used the IPPA scale, Lin et al. [33] used both the Work-Related Stress Scale (WRS) and SAS (both were included in quantitative analysis).

Included studies adopted different Yoga intervention in treatment group. Indeed, Axén et al. [31] used medical Yoga (MY) training sessions, Bhandari et al. [35] used Yogic intervention, Hartfiel et al. [34] and Harfiel et al. [36] used both Dru Yoga, Lin et al. [33] used 60-minutes weekly Yoga classes and Wolever et al. [32] used a Viniyoga stress reduction program.

Data of included studies and their main characteristics are reported in Table 1.

### 3.3. Risk of Bias Assessment

**Figure 3:**
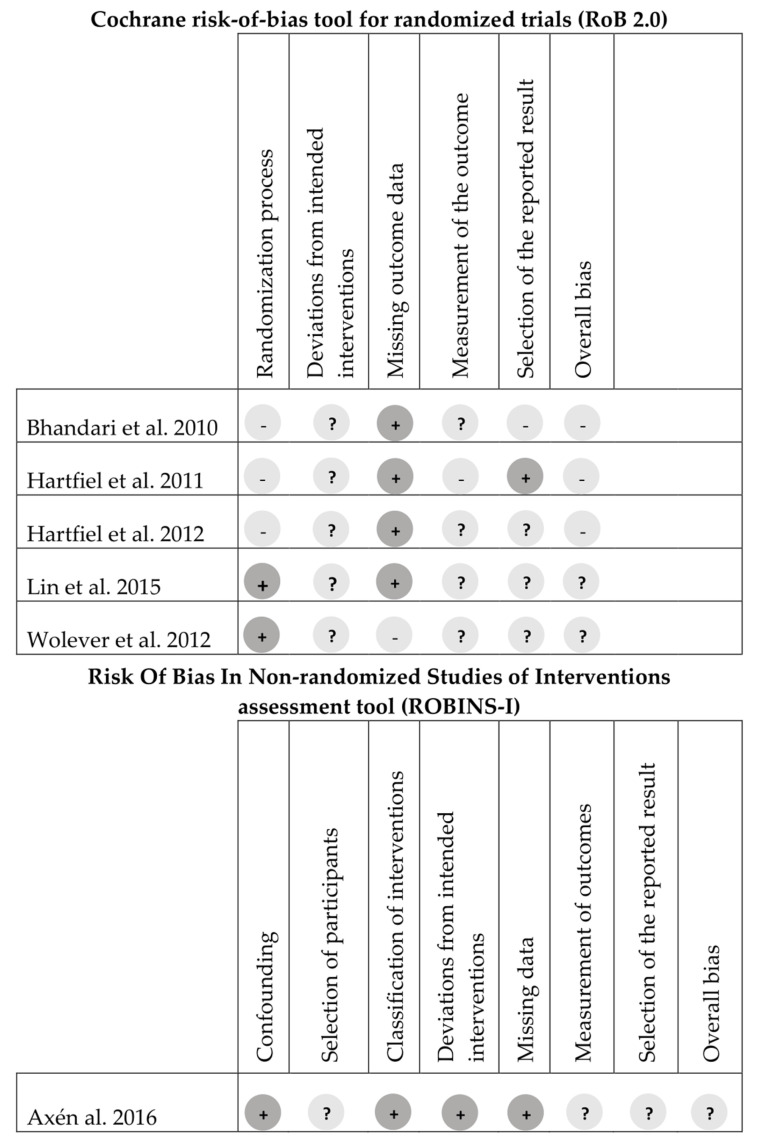
The assessment of the risk of bias of included studies. Note: “+” = low risk, “−“ = high risk, “?” = some concerns.

The studies included in the qualitative synthesis and quantitative analysis had different designs. Indeed, 5 out of 6 of the included studies were randomized clinical trials and 1 [31] was a non-randomized quasi-experimental study. Therefore, risk of bias was assessed through different tools (as reported in methods section) and the results are summarized in Figure 3.

The randomized clinical trials included were considered to suffer from “some concerns” about risk of bias by both assessors (S.P. and I.A.) (between-authors agreement: 100%), and the third author agreed about this judgment. Studies of Lin et al. [33] and Wolever et al. [32] reported a detailed procedure of randomization and allocation concealment and were considered at low risk of bias about these domains. By contrast, Hartfiel et al. [36], Hartfiel et al. [34] and Bhandari et al. [35] have not reported enough data to assess these domains and were considered at “high risk” of bias. All randomized clinical trials aroused “some concerns” about the domain of deviation from intended interventions. Moreover, almost all of studies had data outcome available for nearly all participants, except for Wolever et al. [32].

### 3.4. Quantitative Analysis

**Figure 4:**
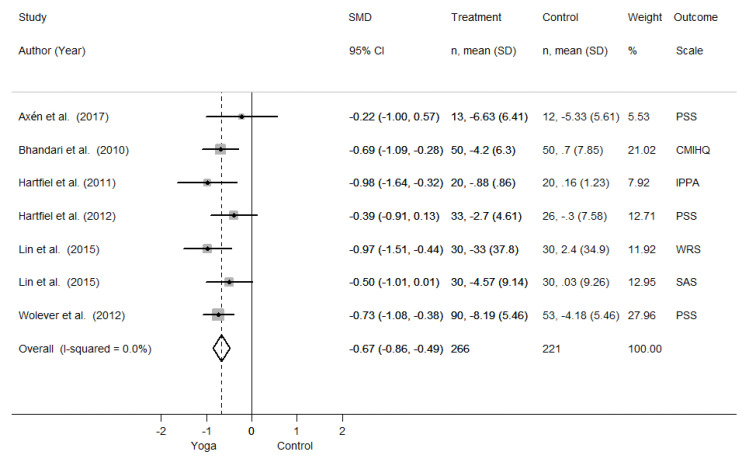
Forest plot reporting quantitative analysis. SMD = Standardized Mean Difference. CI = Confidence Interval. SD = Standard Deviation. PSS = Perceives Stress Scale. CMIHQ = Cornel Medical Index Health Questionnaire, IPPA = Inventory of Positive Psychological Attitudes, WRS = Work-Related Stress Scale, SAS = Stress Adaption Scale.

Quantitative analysis of results is reported in Figure 4. A significant effect in favor of Yoga interventions carried out at workplace in reducing perceived stress among employees is evident (ES: −0.67 [95% confidence interval (CI): −0.86, −0.49]).

The analysis of the results reveals a very low degree of heterogeneity. Indeed, the point estimates of all included studies are in favor of Yoga interventions for the reduction of perceived stress, compared to a control group. Although in the same direction, results from Axén et al. [31], Hartfiel et al. [36] and Lin et al. [33] (only for SAS outcome) are not significant, as their confidence intervals include the null value. This is probably due to the limited sample size and to the uncertainty of the results. Nevertheless, their point estimates are in favor of Yoga too, and the overall interpretation of the pooled results is in favor of Yoga interventions.

Given the similar designs of the included studies and the lack of heterogeneity in results, it was not appropriate to design and conduct further subgroup or sensitive analysis. Moreover, it was impossible to conduct sensitivity analysis based on the type of Yoga proposed as intervention, comparing different form of Yoga among them.

Included studies carried out Yoga interventions in sample of employees with similar work-related demands. Therefore, subgroup analysis based on type of work activity was not appropriated too.

Due to the inclusion of less than 10 studies [37], publication bias was not evaluable by the funnel plot.

## 4. Discussion

The results of the present study allow us to discuss some issues about the effectiveness of Yoga interventions carried out at workplaces in improving perceived stress among employees. The quantitative analysis of the available literature systematically reviewed showed improvement in several common stress outcome measures in workers following a Yoga intervention program.

It is interesting to note that these results come from the analysis of studies conducted in different countries, such as Sweden [31], India [35] and Great Britain [34,36], sustaining the idea of an international interest about this topic. Indeed, the work-related stress is a global problem afflicting countries worldwide. To this scope, several international agencies try to estimate the size of this condition and its direct and indirect consequences, i.e., the European Agency for Safety and Health at Work [1] and others. Excessive workload, role uncertainty, lack of support, physical and mental violence from colleagues or other people, bullying, harassment, unwanted sexual attentions and conflicts within workplaces have been identified as major psychosocial risk factors able to promote work-related stress [1]. Main clinical manifestations of stress could be anxiety, irritability, work-related fatigue, sleep disturbances, distress, and burnout [11]. To manage this health problem is an emerging issue in public health [37,38].

Both genders are affected, with a little higher prevalence among men (22.9% vs. 20.3%); the most affected age range is between 40 and 54 years [39]. These data about age are in line with characteristics of workers enrolled in the present meta-analysis.

The relationship between work-related stress and type of occupation was investigated too. Indeed, some reports highlight that workers in education and health sectors and in agriculture showed the highest level of perceived stress [39]. In other reports, public administration and defense sectors are particularly involved [39]. Other evidence suggests that stress is frequent and particularly important in employees who have an occupation that requires low physical activity [40]. In line with these results, 2 out of 6 studies included in the present study enrolled healthcare personnel [31,33]. Another 2 studies included employees in education [34] and in public administration [36]. The impact of work-related stress among healthcare personnel has received a particular attention in the scientific literature, as this peculiar worksite is commonly exposed to stressful situations determining potential relevant healthy and economic consequences [41,42].

Work-related stress incidence has been almost stable during the last few years and insignificant effects on its reduction have been recorded. Conversely, it has shown slight signs of increasing during the last few years [38].

Often this condition is underestimated and not managed properly [41], leading to an increase of several negative outcomes like a higher risk of cardiovascular diseases [43], stroke, of other chronic diseases like asthma, type 2 diabetes mellitus, metabolic syndrome, and of some specific cancers [44,45].

Although the relationship between work-related stress and incidence of specific forms of cancers seems difficult to investigate, a recent meta-analysis including near 300,000 participants confirms a significantly higher risk ratios for incidence of colorectal, lung, and esophagus cancers among subjects suffering from work-related stress, after controlling for confounders [45]. Molecular mechanisms proposed involved hormone homeostasis, as changes in levels of glucocorticoids and catecholamines [45]. These hormones interfere with different cellular functions, like apoptosis processes and free oxygen radicals generation with an impact on oxidative stress and promotion of tumorigenesis [46,47,48].

Therefore, considering that work-related stress is implicated in numerous health conditions, it is a priority to include a focus on its management to reduce the burden of several diseases.

To treat this condition successfully, it is mandatory to find a valuable method to assess work-related stress. Some physiological parameters, like blood systolic pressure, are difficult to relate to stress in a cause–effect relationship. Others, like hematic level of cortisol, are difficult to carry out on a large scale. Therefore, valid measures of perceived stress represent a common method to self-judge this problem from a workers’ point of view. To this end, several questionnaires and evaluation scales are adopted. One of the most commonly used (as seen in Axén et al. [31], Harfiel et al. [36] and Wolever et al. [32]) is the Perceived Stress Scale: this is a 10-items scale measuring the extent to which a person perceives life situations as stressful, and collecting data on feelings and thoughts during the last month [49]. By contrast, in the present study, Lin et al. [33] used a work-related stress scale derived by a Chinese version by Chang [50], with 30 questions referring to stressful experiences during the last month and general distress. Commonly, other methods adopted by included studies, like IPPA or SAS, are poorly used. To the authors’ best knowledge, no previous studies were carried out to compare methods to assess perceived stress among workers, adopting different evaluation scales. Data of the present study confirm that the PSS is the most widely adopted method, but this does not imply that it is also the most valuable. Therefore, future studies could be useful to assess the validity of different methods to measure the “amount” and the impact of stress among workers, defining better the size of the problem and identifying workers at high risk of work-related stress able to induce other health manifestations.

Interestingly, all included studies used different Yoga programs. For example, Hartfiel et al. [34] used Dru Yoga classes: these consist of flowing move ments, directed breathing, and relaxation techniques that included affirmation and visualization [51]. Wolever et al. [32] used the Viniyoga stress reduction program, a 12-week (12-hour) program including physical postures, breathing techniques, guided relaxations, and mental techniques [52]. Therefore, differences in Yoga styles exist and future research could aim to evaluate if different forms of Yoga could have different effectiveness on work-related stress reduction.

Nevertheless, beyond differences in the method of included studies, the key questions and designs were similar, and it was possible to pool their results quantitatively. Indeed, these results represent the summary of findings about Yoga interventions carried out at workplaces, as part of corporate wellness programs, to reduce work-related stress among employees.

Admittedly, the present meta-analysis suffers from some limitations. A major one is the small number of included studies with high or unclear risk of bias, assessed using Cochrane’s tool. For these reasons, further well-designed studies are mandatory. Moreover, characteristics of enrolled patients in the included studies have not been reported extensively: this issue could compromise the pooling of the results apparently, although ensuring a high degree of external validity. Furthermore, included studies used different protocols of Yoga, with different duration and style: but no guidelines exist regarding the frequency and the type of this practice, as before discussed. Additionally, different outcome measures for stress have been used in included studies: although these are subjective methods to quantify stress levels, their scientific validity has already been proved.

Nevertheless, beyond these unavoidable limitations that could reduce the scientific relevance of the results, it is necessary to analyze the problem in a wider point of view. Indeed, based on these available results, several considerations could be included to plan practical activities and to design future studies confirming the effectiveness of Yoga interventions for work-related stress reduction.

Like other complementary treatments based on gentle movements and relaxation (such as Tai Chi, Qi Gong, and others) that have showed cognitive and physiological benefits [53] in healthy subjects and in several pathological conditions [54,55], the beneficial mental effects of Yoga and its ability to reduce stress levels have also been recognized [56]. Dalgas et al. [57] proved that Yoga has relevant effects on cognitive functions, endocrine regulations, and other physiological factors; therefore, it should be effective in improving depression and mental disorders. Regular practice of Yoga promotes strength, endurance, flexibility and facilitates characteristics of friendliness, compassion, and greater self-control [58]. Moreover, as seen in Wolever et al. [32], it improves sleep difficulties, breathing rate and heart rhythm.

Therefore, the effectiveness of Yoga as a recreational activity or complementary medicine is widely proved in the general population. Recent studies have started to analyze its effectiveness in corporate wellness program carried out at workplaces [56,59], confirming that Yoga has a positive effect on health, and it is a valuable method to reduce work-related stress.

Nevertheless, the results of the present study add a quantitative evidence in favor of Yoga interventions. The statistical pooling of the results through a meta-analysis of intervention is a valuable method to establish stronger scientific evidence. Obviously, based on inclusion criteria of the present study, Yoga interventions have been compared to a control group, namely vs. a no-treatment intervention. No data are available about the superiority or inferiority of Yoga compared to other stress-reduction programs, i.e., others mindfulness activities or meditative approaches.

To investigate this problem scientifically, several other studies are needed that are able to summarize available literature. For example, meta-analytic investigation of studies comparing Yoga interventions to other treatments should be useful to choose the most valuable activity to reduce stress at workplace. Modern statistical approaches have extended meta-analysis beyond comparisons of intervention. Network meta-analysis could be relevant to compare more than 2 treatments about stress management programs at workplaces [60,61]. Meta-analyses of prevalence are commonly conducted in several fields of medical scientific literature and could be particularly useful in occupational medicine [62,63]. The real worldwide prevalence of work-related stress could be assessed through this methodology. Even the pooled prevalence of companies administrating stress-reduction programs, the pooled prevalence of workers participating in Yoga interventions programs, or the pooled prevalence of reasons behind lost working-days could be explored by this methodology in future studies.

Therefore, the results of the present study confirm the effectiveness of Yoga as a method to manage work-related stress, although some limitations in included studies exist, and justify the rationale for further investigations.

## 5. Conclusions

Work-related stress is a complex problem in occupational medicine, related to several psychosocial risk factors. A higher level of perceived stress among workers could contribute to determining health problems and promote the onset of some cardiovascular, musculoskeletal, and other diseases. Therefore, it is necessary to design, plan and conduct work-related stress-reduction programs. A valuable setting to perform these activities is the workplace and one of the most adopted method is represented by Yoga interventions carried out at workplaces directly, as part of corporate wellness programs. The synthesis of the available evidence and its quantitative analysis prove the effectiveness of Yoga interventions carried out at workplaces in decreasing perceived stress among employees, when compared to no-treatment. Nevertheless, these conclusions are based on a few studies with “some concerns” about methodological rigor and future studies are needed. Indeed, in order to define common characteristics of Yoga interventions (such as style, duration, volume and frequency), and to assess the effectiveness of Yoga interventions compared to other similar approaches, it is mandatory to design effective strategies of research about health programs carried out as part of corporate wellness programs to manage work-related stress.

## Figures and Tables

**Table 1:** Characteristics of included studies.

Author	Year	Country	Study Design	Workplace	Sample Size and Demographic Characteristics	Intervention	Control	Stress Outcome Measures	Conclusion
Axén, et al. [31]	2017	Sweden	Quasi-experimental pilot study	Medical staff in public nursing home and home care services	25; 51 ± 8.9 aged; only 1 man (in which group not specified)	n; age; gender: 13; 54 ± 8.1 aged. Gender not specified Type: Medical Yoga training: breathing exercises, seated exercises, seated meditation Frequency and Volume: 9 weeks, 3 times/week (one at work and two at home)	n; age; gender: 12; 47.5 ± 8.8 aged. Gender not specified Type: NA Frequency and Volume: NA	Perceived Stress Scale (PSS)	No statistical differences between groups; changes in perceived stress were found to correlate statistically to changes in perceived work ability. Convincing unit managers to let their employee participate in this intervention was difficult
Bhandari, et al. [35]	2010	India	Randomized Controlled Trial	Telephone Industry	100; 51 ± 5.5 aged; 50 male and 50 females	n; age; gender: 50; 51 ± 5.5 aged; 25 male and 25 females Type: Yogic interventions: selected postures, Panayams, concentrations and meditations Frequency and Volume: 4 weeks, daily (one hour per day - 6:30–7.30 am - except Sunday)	n; age; gender: 50; 51 ± 5.5 aged; 25 male and 25 females Type: NA Frequency and Volume: NA	Cornel Medical Index Health Questionnaire (CMIHQ-Hindi Versions for male and female)	Significant effect of the yogic intervention to manage distress and enhance work performance
Hartfiel, et al. [34]	2011	UK	Randomized Controlled Trial	British university employees	40; 39.3 aged; 4 male and 36 females	n; age; gender: 20; 40.6 ± 11.40 aged; 3 male and 17 females Type: Dru Yoga: flowing movement, directed breathing and relaxation techniques that included affirmation and visualization Frequency and Volume: 6 weeks, at least one of three 60-minute lunchtime classes per week + guided 35-minute home practice session	n; age; gender: 20; 38 ± 9.58 aged; 1 male and 19 females Type: NA Frequency and Volume: NA	Inventory of Positive Psychological Attitudes (IPPA)	A 6-week program of Yoga had substantial positive effects on the emotional well-being and resilience to stress
Hartfiel, et al. [36]	2012	UK	Randomized Controlled Trial	British local government authority employees	59; aged 25-64 years; 6 male and 53 females	n; age; gender: 33; 46.1 ± 11.5 aged; 4 male and 29 females Type: Dru Yoga: flowing movement, directed breathing and relaxation techniques that included affirmation and visualization Frequency and Volume: 8 weeks, 50 minutes of session each week + 20 minutes DVD for home practice, twice a week	n; age; gender: 26; 43.6 ± 11.5 aged; 2 male and 24 females Type: Frequency and Volume: NA	Perceived Stress Scale (PSS)	An 8-week program of Yoga resulted in significant reduction in stress and back pain, and improved psychological well-being
Lin, et al. [33]	2015	Taiwan	Single-blind, parallel-arm, Randomized Controlled Trial	Mental Health professionals in a teaching hospital	60; 30 years average aged; 12 male and 48 females	n; age; gender: 30; 32.07 ± 7.54 aged; 4 male and 26 females Type: Yoga classes: slower warm-up exercises, forced abdominal breathing, meditation, bodily stretching positions Frequency and Volume: 12 weeks, 60 minutes of session each week	n; age; gender: 30; 29.77 ± 6.89 aged; 8 male and 22 females Type: Free teatime in which they watched television and did not exercise Frequency and Volume: NA	Work-related stress scale derived from the Chinese version of work-related stress scale by Lan	The professionals in the Yoga group experienced a significant reduction in work-related stress and a significant enhancement of stress adaption
Wolever, et al. [32]	2012	USA	Randomized Controlled TrialMulticentric trial with more than 2 arms: Yoga, Mindfulness (at work and online) and Control	Employees of a national insurance carrier	143; 42.9 average aged; 23.4% male	n; age; gender: 90; 41.6 ± 10.1 aged; 24% male Type: Viniyoga Stress Reduction program: asanas, breathing techniques, guided relaxation, mental techniques and education about starting a home practice Frequency and Volume: 12 weeks, 1 hour per week	n; age; gender: 53; 42.7 ± 9.7 aged; 18.9% male Type: Did not receive any stress management intervention. Frequency and Volume: NA	Perceived Stress Scale (PSS)	Yoga intervention and Mindfulness intervention showed positive results compared to control group about stress reduction. No differences were detected comparing at-work vs. online mindfulness programs.
